# The Nucleoid Proteins Fis and IHF Positively Regulate the Gene Expression of Operons Responsible for Producing the Cytotoxins Tilimycin and Tilivalline in *Klebsiella oxytoca*

**DOI:** 10.1155/ijm/2094815

**Published:** 2025-10-13

**Authors:** Fernando Chimal-Cázares, Diana Rodríguez-Valverde, Jessica Martínez-Cruz, Ricardo González-Ugalde, Alan E. Jiménez, Santa Mejía-Ventura, Félix Matadamas-Martínez, Roberto Rosales-Reyes, Sandra Rivera-Gutiérrez, Javier Torres, Steven D. Goodman, James G. Fox, Jorge Soria-Bustos, Miguel A. Ares

**Affiliations:** ^1^Unidad de Investigación Médica en Enfermedades Infecciosas y Parasitarias, Hospital de Pediatría, Centro Médico Nacional Siglo XXI, Instituto Mexicano del Seguro Social, Mexico City, Mexico; ^2^Instituto de Ciencias de la Salud, Universidad Autónoma del Estado de Hidalgo, Tulancingo, Hidalgo, Mexico; ^3^Unidad de Medicina Experimental, Facultad de Medicina, Universidad Nacional Autónoma de México, Mexico City, Mexico; ^4^Departamento de Microbiología, Escuela Nacional de Ciencias Biológicas, Instituto Politécnico Nacional, Mexico City, Mexico; ^5^Center for Microbial Pathogenesis, Abigail Wexner Research Institute at Nationwide Children's Hospital, Department of Pediatrics, College of Medicine, The Ohio State University, Columbus, Ohio, USA; ^6^Division of Comparative Medicine, Massachusetts Institute of Technology, Cambridge, Massachusetts, USA; ^7^Consejo de Ciencia, Tecnología e Innovación de Hidalgo, Hidalgo, Mexico

**Keywords:** cytotoxicity, Fis, gene regulation, IHF, *Klebsiella oxytoca*, tilimycin, tilivalline

## Abstract

**Introduction:**

*Klebsiella oxytoca* causes antibiotic-associated hemorrhagic colitis due to the production of the enterotoxins tilimycin and tilivalline. These toxins are synthesized by enzymes encoded in the *aroX* and NRPS operons, which are expressed divergently. This study investigated how the nucleoid-associated proteins Fis and IHF regulate these operons and influence the production of enterotoxins.

**Methods:**

We used reverse transcription quantitative PCR (RT-qPCR) to assess the role of Fis and IHF in the transcription of the *aroX* and NRPS operons. Electrophoretic mobility shift assays (EMSAs) were used to examine the binding of Fis and IHF to the regulatory region. Additionally, Caco-2 viability assays were performed using cells infected with WT, mutant, and complemented strains.

**Results:**

RT-qPCR demonstrated that deletions of *fis* or *ihfA*/*ihfB* significantly reduced operon expression. EMSA confirmed that Fis and IHF bind specifically to the regulatory region between the *aroX* and NRPS operons. Viability assays in Caco-2 epithelial cells indicated increased host cell survival when exposed to the deletion mutants. Genetic complementation restored both transcription levels and cytotoxicity.

**Conclusions:**

Fis and IHF are positive regulators of the *aroX* and NRPS operons, enhancing the production of tilimycin and tilivalline. These findings highlight the potential of targeting Fis and IHF for therapeutic intervention in antibiotic-associated hemorrhagic colitis.

## 1. Introduction

*Klebsiella oxytoca*, a gram-negative *Bacillus* typically found in the gut microbiota, poses a significant pathogenic threat due to its resistance to penicillins, which is linked to intrinsic antimicrobial resistance genes that produce beta-lactamases [1, 2]. The elimination of other gut microbiota following penicillin treatment leads to dysbiosis, characterized by a marked decrease in commensal and symbiotic microorganisms and an increase in pathobionts such as toxigenic *K. oxytoca*. This disruption has profound implications for the intestinal microbiota and raises serious concerns regarding gut health and stability [3, 4].

The proliferation of toxigenic *K. oxytoca* in the large intestine notably increases the production of the cytotoxins tilimycin (TM) and tilivalline (TV) [5, 6]. TM and TV are nonribosomal peptide enterotoxins produced by enzymes encoded by the divergent *aroX* and NRPS operons located within a pathogenicity island. The *aroX* operon comprises the genes *aroX*, *dhbX*, *icmX*, *adsX*, and *hmoX*, while the NRPS operon consists of *npsA*, *thdA*, and *npsB* [6–8]. Notably, TM induces DNA damage, causing a halt in the cell cycle at the G1/S phase, whereas TV disrupts tubulin function, obstructing microtubule disassembly and halting the cell cycle at G2/M [6]. Moreover, both toxins instigate apoptosis [6], compromising the intestinal barrier in colonic epithelial cells. This disruption can result in antibiotic-associated hemorrhagic colitis (AAHC), manifested by symptoms such as bloody diarrhea, abdominal cramps, and rectal sparing [8–10].

The nucleoid-associated proteins Fis (factor for inversion stimulation) and IHF (integration host factor) play a significant role in organizing bacterial chromosomes and governing gene expression. Fis functions as a homodimeric protein that is crucial for facilitating DNA bending and overseeing transcription, which encompasses both activation and repression. Its roles are essential for various processes, including virulence, metabolism, and stress responses, positioning Fis as a key player during bacterial proliferation, where regulating adaptive and survival-related genes is critical [11–13]. In contrast to Fis, IHF is a heterodimeric protein consisting of both an alpha and a beta subunit, which specifically binds to DNA sequences and creates the sharp bends necessary for DNA compaction, replication, recombination, and precise tuning of gene expression [14].

In our prior research, we demonstrated that the transcription of the *aroX* and NRPS operons is effectively activated by the global regulator CRP (cAMP receptor protein), with the process significantly enhanced by lactose [15]. Additionally, we identified that the global regulator Lrp (leucine-responsive regulatory protein) is crucial for the activation of these operons, a process further intensified by leucine [16]. Conversely, our results also indicate that the global response regulator OmpR represses the transcription of the *aroX* and NRPS operons [17]. While these studies highlight the involvement of several global regulators, the contribution of other factors, particularly nucleoid-associated proteins, in modulating the expression of the *aroX* and *NRPS* operons remains underexplored.

This study demonstrates that nucleoid proteins Fis and IHF enhance the transcription of the *aroX* and NRPS operons by binding to their regulatory regions. Additionally, the impact on Caco-2 cells reveals that, without these proteins, the cytotoxic effect is reduced, leading to increased cell viability. This underscores their crucial role as activators in TM/TV biosynthesis and the pathogenicity of toxigenic *K. oxytoca*.

## 2. Materials and Methods

### 2.1. Bacterial Strains and Growth Conditions

We utilized the toxigenic strain *K. oxytoca* MIT 09-7231 [18], along with its derivative mutants (Δ*fis*, Δ*ihfA*, and Δ*ihfB*) and complemented strains (Δ*fis*::*fis*, Δ*ihfA* pT3-IHF*α*, and Δ*ihfB* pT3-IHF*β*). A complete list of the strains and plasmids used in this study can be found in Table 1. The cultures were grown in Tryptone Soy Broth (TSB) (Difco, Beirut, Lebanon) at 37°C. Antibiotics were added as necessary: ampicillin (200 *μ*g/mL), kanamycin (50 *μ*g/mL), or tetracycline (10 *μ*g/mL). Experiments with all *K*. *oxytoca* strains were conducted using cultures at the mid-exponential growth phase, with an OD_600nm_ of 0.4.

### 2.2. Generation of the Δ*fis*, Δ*ihfA*, and Δ*ihfB* Mutant Strains

The *fis*, *ihfA*, and *ihfB* genes were deleted using a one-step mutagenesis approach, where each target gene was replaced with a kanamycin resistance cassette through the *λ*-Red recombinase system, as previously described [21]. Briefly, PCR fragments containing the *fis*, *ihfA*, and *ihfB* sequences flanking the kanamycin resistance gene were amplified using gene-specific primer pairs (Table 2). These purified PCR products were then electroporated into competent *K. oxytoca* cells that contained the *λ*-Red recombinase helper plasmid pKD119. The expression of the recombinase system was induced by adding L-(+)-arabinose (Sigma, St. Louis, MO, United States) to a final concentration of 1%. The successful generation of each mutant strain was verified through PCR and sequencing to ensure the accuracy of the gene deletions.

### 2.3. Plasmid Construction

The plasmids pT3-IHF*α* and pT3-IHF*β* were engineered to complement the Δ*ihfA* and Δ*ihfB* mutant strains, respectively. The *ihfA* and *ihfB* genes from *K. oxytoca* were amplified via PCR using gene-specific primers (Table 2) and cloned into the pMPM-T3 vector using *Xho*I and *Eco*RI restriction sites. To produce the Fis recombinant protein, the *fis* gene included a nucleotide sequence encoding a His_6_-tag at its C-terminus. This *fis-*His_6_ gene was inserted into the pMPM-T6 vector using *Nco*I and *Hin*dIII restriction sites, resulting in the pT6-Fis plasmid. All constructed plasmids were confirmed by DNA sequencing.

### 2.4. Generation of *Cis*-Complemented Δ*fis::fis* Strain

To successfully generate the *cis*-complemented Δ*fis*::*fis* strain according to the previously established protocols [22, 23], we first removed the FRT-flanked kanamycin resistance cassette from the Δ*fis* mutant strain by transforming it with the pCP20 plasmid, following the established protocol [21]. Next, we amplified the *fis* gene from *K*. *oxytoca* using PCR, purified it, and cloned it into the p2795 vector using *Bam*HI and *Xho*I restriction sites. The correct construction of the plasmid was confirmed through DNA sequencing.

We then created a DNA fragment that contained the *fis* gene fused to a kanamycin resistance cassette at its 3⁣′ end using specific primers (Table 2). These primers included 45-nucleotide sequences homologous to the essential 5⁣′ and 3⁣′ ends of the *K*. *oxytoca fis* gene. After purification with a column (Qiagen, Hilden, Germany), we electroporated the resulting fragment into *K*. *oxytoca* Δ*fis*::FRT cells carrying the helper plasmid pKD119. To effectively induce the expression of the *λ*-Red recombinase system, we added L-(+)-arabinose (Sigma, St. Louis, MO, United States) to a final concentration of 1%. After incubating at 37°C, we selected transformants resistant to kanamycin and conducted a comprehensive analysis using PCR and DNA sequencing. This confirmed the successful integration of the *fis* gene into the target genomic locus.

### 2.5. RNA Isolation and Reverse Transcription Quantitative PCR (RT-qPCR)

RNA was extracted from bacterial cultures during the mid-exponential growth phase (OD_600nm_ of 0.4) using the hot phenol method [24]. The extracted RNA was treated with the TURBO DNA-free kit (Invitrogen, Waltham, MA, United States) to remove contaminating DNA. The concentration and purity of the RNA were assessed using a NanoDrop ONE spectrophotometer (Thermo Scientific, Waltham, MA, United States), and RNA integrity was evaluated by electrophoresis on a bleach-denaturing 1.5% agarose gel [25].

For cDNA synthesis, we utilized 1 *μ*g of purified RNA as a template with the RevertAid First Strand cDNA Synthesis Kit (Thermo Scientific, Waltham, MA, United States), strictly adhering to the manufacturer's instructions. Control reactions without reverse transcriptase were included in each experiment to effectively eliminate any possibility of genomic DNA contamination. Quantitative PCR (qPCR) was performed on a LightCycler 480 instrument (Roche, Basel, Switzerland) using SYBR Green I Master Mix (Roche, Basel, Switzerland) and gene-specific primers (Table 2). Each reaction mixture contained 1.5 *μ*L of PCR-grade water, 0.5 *μ*L (10 *μ*M) of forward primer, 0.5 *μ*L (10 *μ*M) of reverse primer, 5 *μ*L of 2X SYBR Green I Master Mix, and 2.5 *μ*L of cDNA (~25 ng). Samples were loaded into a multiwell plate and amplified in triplicate across three independent experiments. The *rrsH* gene (16S rRNA) served as an internal reference for normalization.

The qPCR protocol included (1) an initial denaturation step at 95°C for 10 min; (2) 45 amplification cycles consisting of 10 s at 95°C, 10 s at 59°C, and 10 s at 72°C, with fluorescence measured at each cycle; (3) a melting curve analysis beginning at 95°C for 10 s, followed by 1 min at 65°C with continuous fluorescence measurement up to 97°C; and (4) a final cooling step at 40°C for 10 s.

To ensure the absence of DNA contamination, RNA samples without reverse transcription were subjected to qPCR, and no amplification was observed after 45 cycles. Additionally, reverse transcriptase-minus controls were included in all experiments. Gene expression fold changes were calculated using the 2^−ΔΔCt^ [26, 27], ensuring robust and accurate quantification of transcriptional changes.

### 2.6. Purification of Fis Protein

The Fis-His_6_ expression vector, pT6-Fis, was introduced into competent *Escherichia coli* BL21 (DE3) cells via electroporation, followed by purification using an optimized protocol. Bacteria containing the recombinant plasmid were cultured to mid-exponential phase, and the expression of Fis-His_6_ was induced with 1% L-(+)-arabinose at 37°C for 6 h. Cells were centrifuged, and the pellet was resuspended in a buffer consisting of 8 M urea, 100 mM Na_2_HPO_4_, and 10 mM Tris-HCl (pH 8.0). After sonication, debris was removed by centrifugation, and the supernatant was filtered and applied to a Ni-NTA agarose column (Qiagen, Hilden, Germany) for affinity purification.

The column was washed with 200 mL of washing buffer (50 mM imidazole, 10 mM Na_2_HPO_4_, 1.8 mM KH_2_PO_4_, 137 mM NaCl, and 2.7 mM KCl at pH 7.4). Fis-His_6_ was then eluted using 10 mL of elution buffer (500 mM imidazole, 10 mM Na_2_HPO_4_, 1.8 mM KH_2_PO_4_, 137 mM NaCl, and 2.7 mM KCl at pH 7.4). Purity was confirmed through SDS-PAGE and Coomassie blue staining, while protein concentration was quantified using the Bradford method (Bio-Rad, Hercules, CA, United States). To ensure stability and activity, the purified Fis-His_6_ protein was stored at −70°C in a buffer containing 50% glycerol, 10 mM Na_2_HPO_4_, 1.8 mM KH_2_PO_4_, 137 mM NaCl, and 2.7 mM KCl at pH 7.4.

### 2.7. Purification of IHF Protein

IHF was purified from *E*. *coli* strain HN880 [28] by culturing the cells at 30°C until an optical density (OD_600nm_) of 0.4–0.6 was achieved. The temperature was then increased to 42°C for 4 h to induce IHF expression. Following cell harvesting via centrifugation at 15,000 × g for 5 min, the cells were resuspended in lysis buffer (20 mM Tris pH 7.4, 1 mM EDTA, 10% glycerol, 20 mM NaCl, 1 mM PMSF, 5 mM MgCl_2_, 5 mM CaCl_2_).

The suspension was treated with DNase (100 *μ*g/mL) on ice for 30 min and then lysed using a French press at 20,000 psi. The lysate was clarified by centrifugation at 25,000 × g for 30 min at 4°C and filtered through a 0.45 *μ*m membrane. Proteins were precipitated with ammonium sulfate to 50% saturation, centrifuged again, and further precipitated to 80% saturation. The proteins were then resuspended in binding buffer and dialyzed overnight at 4°C.

The sample was applied to a heparin-Sepharose column, and IHF was eluted using a linear gradient of elution buffer (10 mM phosphate buffer, pH 7.0, 2 M NaCl). Fractions containing IHF were pooled, analyzed by SDS-PAGE, and purified using a Co-Talon column (GE Healthcare, Chicago, IL). Purified IHF protein was eluted with 10 mM Tris (pH 7.4) and 300 mM KCl. Finally, the protein was concentrated and dialyzed into a storage buffer (50 mM Tris pH 7.4, 600 mM KCl, 1 mM EDTA, 10% glycerol) before being stored at −80°C.

### 2.8. Electrophoretic Mobility Shift Assays (EMSAs)

We used a 448-bp DNA fragment from the regulatory intergenic region located between the divergent *aroX* and *npsA* genes, applying it at a concentration of 100 ng. This DNA probe was carefully incubated with purified proteins, Fis (ranging from 0.0 to 1.0 *μ*M) and IHF (from 0.0 to 0.1 *μ*M), in a specifically formulated binding buffer comprising 1X H/S solution (40 mM HEPES, 8.0 mM MgCl_2_, 50 mM KCl, 1.0 mM DTT, 0.05% NP40, and 0.1 mg/mL BSA). To confirm the specificity of our results, we included a negative control probe from the coding region of the *Mycobacterium tuberculosis fbpA* gene.

The reaction mixtures were meticulously incubated at room temperature for 20 min to promote effective binding before loading onto 6% nondenaturing polyacrylamide gels. Electrophoresis was conducted in a 0.5X Tris-borate-EDTA (TBE) buffer to enable high-resolution separation of the complexes. Following this, the gels were stained with ethidium bromide, allowing us to visualize the DNA-protein complexes under UV light, thus affirming the binding interactions with clarity and precision.

### 2.9. Promoter Prediction in the *aroX*–*npsA* Intergenic Region

To identify putative promoters upstream of *aroX* and *npsA*, we examined a 430-bp sequence located before the start codon of each gene. The genomic sequence of *K. oxytoca* MIT 09-7231 (GenBank Accession No. GCA_001078175.1) was analyzed using the BPROM tool available through the Softberry web software (http://www.softberry.com/berry.phtml?topic=bprom%26group=programs%26subgroup=gfindb).

### 2.10. Cell Viability of Caco-2 Cells

Caco-2 cell viability was calculated using the resazurin reduction assay [29]. Caco-2 cells were plated at a density of 7 × 10^4^ cells per well in 96-well flat-bottom microplates. The cells were cultured in Dulbecco's Modified Eagle's Medium (DMEM) (Thermo Fisher Scientific, Waltham, MA, United States) supplemented with 10% heat-inactivated fetal bovine serum (FBS), 100 U/mL of penicillin, and 100 *μ*g/mL of streptomycin (Invitro, Mexico City, Mexico). To allow the cells to adhere, the plates were incubated for 24 h at 37°C in a 5% CO_2_ atmosphere. After this initial incubation period, the culture medium was replaced with 100 *μ*L of bacterial supernatant and 100 *μ*L of fresh DMEM containing 10% FBS. The cells were then incubated for an additional 48 h. All experiments were conducted in triplicate.

Following incubation, 20 *μ*L of a 2.5 mM resazurin solution was added to each well, and the plates were incubated for an additional 4 h. A negative cytotoxicity control, consisting of Caco-2 cells treated with 200 *μ*L of TSB culture medium, was included. Additionally, background control wells containing only 200 *μ*L of TSB culture medium without cells were prepared. The fluorescence intensity of resorufin, the reduced form of resazurin, was measured using a fluorometer (Fluoroskan Ascent, Thermo Fisher Scientific, Waltham, MA, United States) at an excitation wavelength of 544 nm and an emission wavelength of 590 nm.

The percentage of viable cells was calculated according to the following formula:

 $$\begin{array}{c} \text{Cell viability }\left(\%\right)=\frac{{\text{Fluorescence}}_{\text{sample}}-{\text{Fluorescence}}_{\text{background}}}{{\text{Fluorescence}}_{\text{negative⁣control}}-{\text{Fluorescence}}_{\text{background}}}\times 100. \end{array}$$

### 2.11. Statistical Analysis

For gene expression and cell viability analysis, experiments were conducted in triplicate across three independent biological replicates. To ensure a thorough and complete analysis, we performed one-way ANOVA followed by Tukey's post hoc test using GraphPad Prism 10.4 software (GraphPad Inc., San Diego, CA, United States). Values of *p* < 0.05 were considered statistically significant.

## 3. Results

### 3.1. Fis and IHF Activate the Transcription of the aroX and NRPS Operons

**Figure 1:**
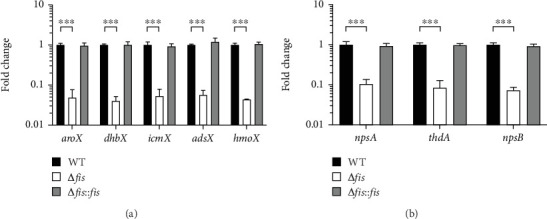
Regulation of transcription by the Fis nucleoid protein on *aroX* and NRPS operons. The transcriptional expression of (a) *aroX* and (b) NRPS operons in the WT, mutant (Δ*fis*), and *cis*-complemented (Δ*fis*::*fis*) strains is shown. The data represent mean values from three independent experiments, each conducted in triplicate, along with corresponding standard deviations. Statistical significance is indicated as follows: ⁣^∗∗∗^*p* < 0.001. All *p* values were calculated using one-way ANOVA and Tukey's post hoc test.

The expression levels of the *aroX* and NRPS operons were significantly downregulated in the Δ*fis* mutant strain, thereby demonstrating the critical influence of Fis on gene regulation. The *aroX* operon exhibited a substantial reduction of approximately 20.7-fold, whereas the NRPS operon experienced a decline of approximately 11.6-fold when compared to the wild-type (WT) strain. Notably, in the complemented Δ*fis*::*fis* strain, the transcription levels were completely restored to those observed in the WT strain, thus underscoring the fundamental role of Fis in regulating these operons (Figure 1a,b).

**Figure 2:**
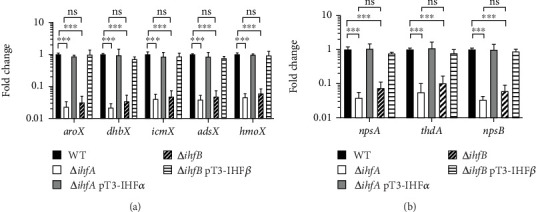
Regulation of transcription by the IHF nucleoid protein on *aroX* and NRPS operons. The transcriptional expression of (a) *aroX* and (b) NRPS operons is shown for WT, mutants (*ihfA*/*ihfB*), and *trans*-complemented (*ihfA* pT3-IHF*α*/*ihfB* pT3-IHF*β*) strains. The data are presented as the mean values from three independent experiments, each performed in triplicate, along with the corresponding standard deviations. Statistical significance is indicated as follows: ⁣^∗∗∗^*p* < 0.001; ns, not significant. All *p* values were determined using one-way ANOVA and Tukey's post hoc test.

The Δ*ihfA* and Δ*ihfB* strains exhibited lower expression levels of the *aroX* and NRPS operons. Specifically, the Δ*ihfA* strain showed a 29.6-fold decrease in *aro*X expression, while the Δ*ihfB* strain had a 28.2-fold reduction compared to the WT. Additionally, the NRPS operon was significantly downregulated, experiencing a reduction of approximately 23.5-fold in Δ*ihfA* and 12.8-fold in Δ*ihfB*. The changes in expression levels for both operons were comparable between the Δ*ihfA* and Δ*ihfB* strains, indicating no statistical differences. In the complemented Δ*ihfA* pT3-IHF*α* and Δ*ihfB* pT3-IHF*β* strains, transcription levels reverted to those of the WT, highlighting the essential role of IHF in regulating these operons (Figure 2a,b).

### 3.2. Fis and IHF Bind to the *aroX* and NRPS Regulatory Regions

**Figure 3:**
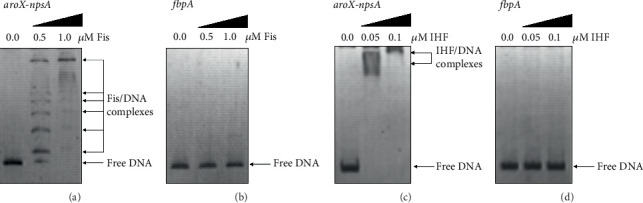
Binding of Fis and IHF to the intergenic region of the *aroX* and *npsA* genes. (a, c) Electrophoretic mobility shift assays (EMSAs) were performed to determine the binding of purified Fis and IHF proteins to a DNA probe from the intergenic regulatory region of the *aroX* and *npsA* genes. (b, d) DNA probes from the *M*. *tuberculosis fbpA* coding region served as negative controls. DNA fragments (100 ng) were individually mixed and incubated with increasing concentrations of purified Fis and IHF proteins. Arrows indicate free DNA, or Fis/DNA and IHF/DNA complexes, stained with ethidium bromide.

This study provides compelling evidence from controlled experiments that elucidate the binding dynamics of Fis and IHF proteins. We employed EMSAs to examine the interactions between recombinant Fis and IHF proteins and various DNA fragments. The *aroX* and NRPS operons are positioned as divergent operons sharing a regulatory intergenic region for gene transcription. Our findings reveal that Fis binds to the regulatory region situated between the divergent *aroX* and *npsA* genes, the first genes in the *aroX* and NRPS operons, at protein concentrations of 0.5 and 1.0 *μ*M (Figure 3a). Additionally, IHF demonstrated substantial binding to the same intergenic region at concentrations of 0.05 and 0.1 *μ*M (Figure 3c).

To verify the specificity of these interactions, we used DNA fragments from the coding region of the *M. tuberculosis fbpA* gene as negative controls for both proteins (Figure 3b,d). These results provide essential insights into the regulatory mechanisms of gene expression that are directly controlled by Fis and IHF proteins.

### 3.3. Enhanced Viability of Caco-2 Cells in the Absence of Fis and IHF

**Figure 4:**
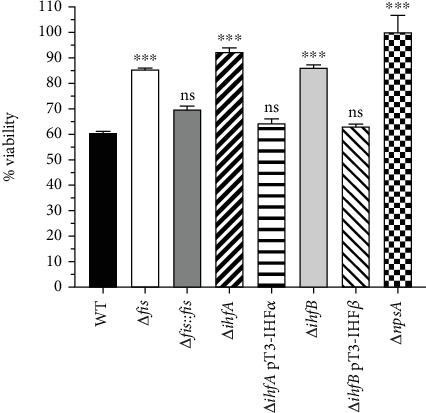
Resazurin-based cell viability assay. Caco-2 cells were treated with supernatants from *K*. *oxytoca* strains (WT, Δ*fis*, Δ*fis*::*fis*, Δ*ihfA*, Δ*ihfA* pT3-IHF*α*, Δ*ihfB*, Δ*ihfB* pT3-IHF*β*, and Δ*npsA*) obtained from cultures with an optical density (OD) of 0.4 at 600 nm. The cells were incubated for 48 h. Following the treatment, cell viability was assessed using the resazurin assay, with fluorescence measurements employed to quantify metabolic activity. The data represent mean values from three independent experiments, each conducted in triplicate, along with standard deviations. Statistical significance is indicated as follows: ⁣^∗∗∗^*p* < 0.001; ns, not significant compared to the WT strain. All *p* values were calculated using one-way ANOVA followed by Tukey's post hoc test.

As expected, the supernatant from the WT strain markedly decreased the viability of Caco-2 cells, confirming the cytotoxic impact of toxigenic *K. oxytoca* on epithelial cells. In contrast, the supernatants from the Δ*fis*, Δ*ihfA*, and Δ*ihfB* strains showed significantly higher cell viability, suggesting a marked reduction in their cytotoxic effects. Notably, when these strains were complemented, their viability returned to levels nearly indistinguishable from those of the WT strain, further validating the reliability of our experimental approach (Figure 4). To elucidate the mechanisms underlying these effects, we analyzed the Δ*npsA* mutant strain, which cannot synthesize the essential TM and TV compounds. As expected, the supernatant from the Δ*npsA* mutant showed no cytotoxicity, resulting in complete viability of Caco-2 cells. This important finding underscores the role of TM and TV compounds in mediating the pathogenic effects of toxigenic *K. oxytoca* on epithelial cells (Figure 4).

### 3.4. Prediction of Putative Promoters and Fis and IHF Binding Motifs on the *aroX*-*npsA* Intergenic Regulatory Region

**Figure 5:**
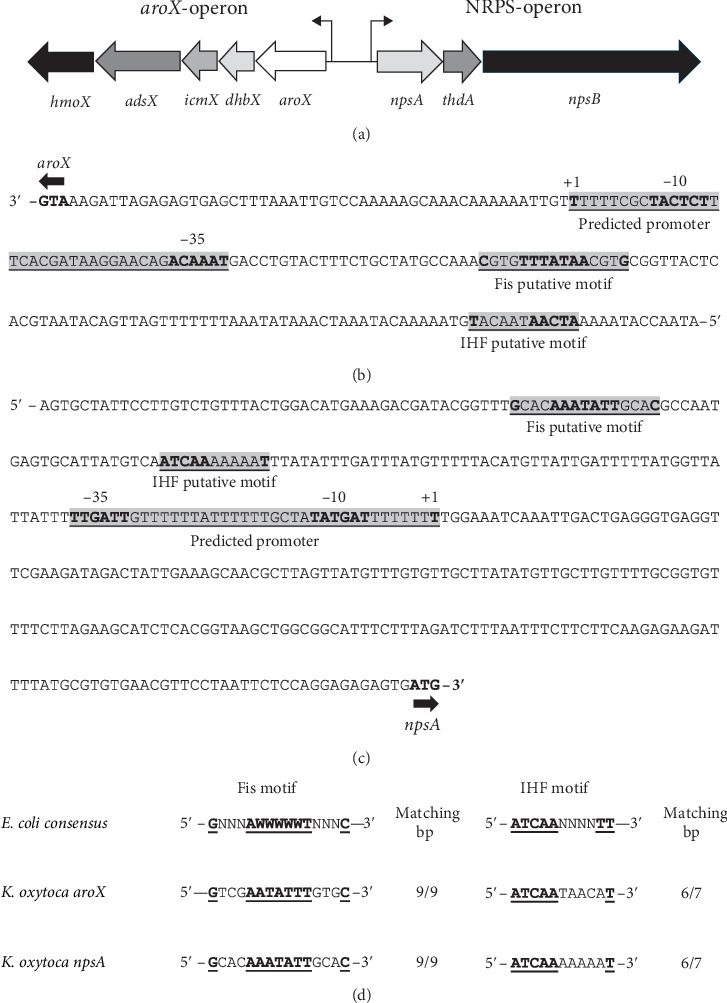
Analysis of the intergenic regulatory region of *aroX* and NRPS operons. (a) The genetic organization of the *aroX* and NRPS operons is depicted. (b) Potential Fis and IHF binding motifs are identified in the regulatory region of *aroX*. (c) The corresponding regulatory region of *npsA* also contains putative binding motifs. Both motifs are underlined and highlighted in gray, with the bases that match the consensus sequence shown in bold. Additionally, for both regulatory regions, a putative promoter was identified through *in silico* analysis, which is highlighted in gray. The putative transcription start site (+1) and the consensus −10 and −35 boxes are indicated in bold. (d) A comparison of the putative Fis and IHF binding motifs in *K. oxytoca* with the consensus sequences from *E*. *coli* is provided. The nucleotides that align with the *E. coli* consensus sequences are underlined and bolded.

The cytotoxin biosynthetic gene cluster is intricately organized into two diverging operons: the *aroX* operon and the NRPS operon (Figure 5a). The *aroX* operon encompasses the genes *aroX*, *dhbX*, *icmX*, *adsX*, and *hmoX*, while the NRPS operon includes the genes *npsA*, *thdA*, and *npsB* [15, 16, 30, 31].

To identify possible regulatory elements, we performed a detailed analysis of the intergenic region between the *aroX* and *npsA* genes. This analysis uncovered two notable putative binding sites for the Fis and IHF regulators in the promoter regions of each operon. For the *aroX* operon, the Fis binding motif was found 112 bp before the start codon, while the IHF binding motif was found 182 bp before the start codon (Figure 5b). In the NRPS operon, the Fis binding motif was identified 330 bp before the start codon, with the IHF binding motif located 298 bp before the start codon (Figure 5c).

Furthermore, by an *in silico* analysis, we identified putative promoters for the *aroX* and *npsA* genes located 50 and 210 bp upstream of their coding regions, respectively (Figure 5). For the *aroX* gene, the predicted Fis binding site was located 61 bp upstream of the putative TSS (+1), and the IHF site was 131 bp upstream (Figure 5). For the *npsA* gene, the Fis binding site was positioned 119 bp upstream of the TSS, while the IHF site was 87 bp upstream (Figure 5).

We further substantiated our findings by comparing the discovered Fis and IHF binding motifs against consensus sequences from *E. coli*. The Fis sequence (GNNNAWWWWWTNNNC) displayed a perfect match, aligning completely with all 9 bp of the *E. coli* consensus sequence [32] for the *aroX* and NRPS operons. Additionally, the IHF sequence (ATCAANNNNTT) aligned with 6 out of 7 bp of the *E. coli* consensus sequence (Figure 5d) [18].

## 4. Discussion

This work examines the contribution of the global regulators Fis and IHF in regulating the expression of the *aroX* and NRPS operons in *K. oxytoca*. These operons encode the enzymatic machinery required for the biosynthesis of the cytotoxins TM and TV, compounds that impair intestinal epithelial integrity and cause host cell damage. Establishing a direct link between Fis and IHF activity and the transcription of these toxin biosynthetic operons provides a basis for understanding how nucleoid-associated proteins integrate environmental inputs, nutrient signals, and host-derived stresses to regulate virulence.

Deletion of *fis* or *ihfA*/*ihfB* reduced transcription of both *aroX* and NRPS operons, as shown by RT-qPCR. The reduction was consistent with earlier studies in other pathogens where Fis and IHF modulate virulence-associated genes [14, 33–35]. In *E. coli*, Fis adjusts gene expression programs in response to nutritional availability and environmental changes, thereby supporting survival during growth transitions [36]. IHF modifies DNA conformation by bending promoter regions, a property that allows it to facilitate transcriptional activation during stress [37]. The correspondence of these patterns across organisms suggests that the regulatory mechanisms in *K. oxytoca* share features with those of other enteric bacteria; however, although similar mechanisms have been described in *E. coli*, our study extends this knowledge by demonstrating that Fis and IHF regulate the *aroX* and NRPS operons controlling cytotoxin production in *K. oxytoca*, which had not been previously reported.

EMSA showed that Fis and IHF bind directly to the intergenic region between *aroX* and *npsA*. This region functions as a shared regulatory site, and binding there suggests a mechanism of direct transcriptional activation through enhanced RNA polymerase recruitment. Intergenic regulation of divergent operons has been described in several bacterial systems [38]. In *Salmonella enterica*, Fis and IHF occupy intergenic regions to regulate operons involved in virulence and stress adaptation by altering DNA conformation [33, 39]. Similar use of intergenic DNA in *K. oxytoca* would provide a way to synchronize the expression of *aroX* and NRPS operons.

Differences in binding affinity between IHF and Fis revealed a tiered regulatory mechanism. IHF bound at low concentrations (0.05–0.1 *μ*M), consistent with a role in initiating transcriptional activation, while Fis required higher concentrations (0.5–1.0 *μ*M), consistent with a role in adjusting transcription once initiation occurred. A similar hierarchy has been described in *Shigella*, where IHF initiates transcription and Fis modulates expression in response to osmotic stress, host temperature, iron limitation, and pH [40, 41]. This type of layered control would allow *K. oxytoca* to adjust toxin biosynthesis in response to environmental and host-derived signals. The absence of binding in negative controls using the *fbpA* gene confirmed the specificity of these interactions.

Functional assays in Caco-2 epithelial cells demonstrated the consequences of these regulatory events. Supernatants from WT *K. oxytoca* cultures containing TM and TV reduced cell viability. In contrast, supernatants from Δ*fis*, Δ*ihfA*, and Δ*ihfB* mutants resulted in greater epithelial survival, consistent with decreased cytotoxin production. Complementation restored cytotoxicity, linking operon regulation by Fis and IHF directly to host cell damage. These observations agree with previous reports identifying TM and TV as the main virulence factors in AAHC [7, 42]. Comparable strategies occur in other pathogens: Fis promotes expression of the pet cytotoxin in enteroaggregative *E. coli* [43], and IHF enhances cholera toxin production in *Vibrio cholerae* [44].

Analysis of promoter sequences identified potential binding motifs for Fis and IHF within the *aroX* and NRPS promoter regions. The conservation of these motifs across different bacterial species suggests that they are stable components of secondary metabolite regulation. This observation highlights the significance of nucleoid-associated proteins in shaping regulatory networks across various taxa. Our findings suggest that the combined actions of Fis and IHF at the *aroX* and NRPS promoters coordinate cytotoxin expression with cellular physiology. Specifically, Fis promotes activation during periods of rapid growth, while IHF sustains or modulates expression as growth slows or stress becomes apparent. This dual control functions as a growth-logic gate, integrating the state of the nucleoid and the growth regime into the regulation of cytotoxin production. The placement of these two growth-responsive global regulators at the promoters aligns with a model in which toxin gene expression is coordinated with the bacterial growth regime and nucleoid state: Fis activates during rapid growth, while IHF maintains or adjusts expression as growth decelerates. Although we did not directly analyze growth-rate dependence in this study, our framework aligns with well-established paradigms of rRNA promoter control and the phase-dependent dynamics of nucleoid-associated proteins.

Together, our findings show that Fis and IHF regulate the *aroX* and NRPS operons by binding to the intergenic region and modulating transcription. This regulation directly influences TM and TV production and defines the cytotoxic potential of *K. oxytoca*. The combined action of Fis and IHF provides a mechanism for aligning virulence factor production with environmental conditions, supporting the ability of *K. oxytoca* to survive and cause disease in the intestinal niche.

## 5. Conclusions

In summary, these findings strongly highlight the essential roles of Fis and IHF in activating the transcription of the *aroX* and NRPS operons, revealing molecular mechanisms that contribute to the pathogenicity of *K. oxytoca*. By demonstrating that Fis and IHF bind to the intergenic regulatory region, we identify a crucial regulatory point that controls the production of the harmful metabolites TM and TV.

Additionally, our results also have significant implications for understanding the role of gut microbiota in both health and disease. Elevated levels of Fis and IHF in *K. oxytoca* would lead to excessive production of TM and TV, potentially disrupting the balance of gut microbiota and resulting in health problems like AAHC [45, 46]. Therefore, Fis and IHF emerge as attractive candidates for further investigation as potential therapeutic targets. Although their direct exploitation in clinical settings has not been demonstrated, exploring ways to interfere with their regulatory activity could offer new avenues to mitigate the harmful effects of *K. oxytoca* in the gut and help restore microbiota balance.

## Figures and Tables

**Table 1:** Bacterial strains and plasmids used in this study.

	**Description**	**Reference**
Strains		
*K*. *oxytoca* WT	Wild-type *K*. *oxytoca* strain MIT 09-7231	[18]
*K*. *oxytoca* Δ*fis*	*K*. *oxytoca* Δ*fis::*FRT	This study
*K*. *oxytoca* Δ*fis*::*fis*	*K*. *oxytoca* Δ*fis*::*fis*-*Kan* (*cis-*complemented)	This study
*K*. *oxytoca* Δ*ihfA*	*K*. *oxytoca* Δ*ihfA::kan*	This study
*K*. *oxytoca* Δ*ihfA* pT3-IHF*α*	*K*. *oxytoca* Δ*ihfA::kan* + pT3-IHF*α*	This study
*K*. *oxytoca* Δ*ihfB*	*K*. *oxytoca* Δ*ihfB::kan*	This study
*K*. *oxytoca* Δ*ihfB* pT3-IHF*β*	*K*. *oxytoca* Δ*ihfB::kan* + pT3-IHF*β*	This study
*K*. *oxytoca* Δ*npsA*	*K*. *oxytoca* Δ*npsA::*FRT	[15]
*E*. *coli* BL21 (DE3)	F^−^*omp*T *hsd*S_B_(r_B_*^−^,* m_B_*^−^) gal dcm* (DE3)	Invitrogen
*E*. *coli* MC4100	Cloning strain F^−^*araD139*Δ(*argF-lac*) *U169 rspL150 relA1 flbB5301 fruA25 deoC1 ptsF25*	[19]
Plasmids		
pMPM-T3	p15A derivative low-copy-number expression vector, *lac* promoter, Tet^R^	[20]
pT3-IHF*α*	pMPM-T3 derivative expressing *ihfA* from the *lac* promoter, Tet^R^	This study
pT3-IHF*β*	pMPM-T3 derivative expressing *ihfB* from the *lac* promoter, Tet^R^	This study
pMPM-T6	p15A derivative expression vector, pBAD (*ara*) promoter, Tet^R^	[20]
pT6-Fis	pMPM-T6 derivative expressing C-terminal Fis-His_6_ from pBAD (*ara*) promoter, Tet^R^	This study
pKD119	pINT-ts derivative containing the lambda-Red recombinase system under an arabinose-inducible promoter, Tet^R^	[21]
pKD4	pANTsy derivative template plasmid containing the kanamycin cassette for lambda-Red recombination, Amp^R^	[21]

Abbreviations: Amp^R^, ampicillin resistance; Kan^R^, kanamycin resistance; Tet^R^, tetracycline resistance.

**Table 2:** Primers used in this study^a^.

**Primer**	**Sequence (5 **⁣′** ➔ 3 **⁣′**)**	**Target gene**
*For gene deletion*		
fis-H1P1	AACACATTGTAAGGATAACTTATGAACAAGACTCAACTGATTGATTGTAGGCTGGAGCTGCTTCG CAAACACTTAACTGATTAGTATCAGTTCATGCCGTATTTTTTCAG CATATGAATATCCTCCTTAG	*fis*
fis-H2P2		
ihfA-H1P1	CATCATTGAGGGATTGAACCTATGGCGCTTACAAAAGCTGAAATGTGTAGGCTGGAGCTGCTTCG GGCCTTTTTAGTTAGTTCAGATTATTTGTCTCTGGGTGACGCATT CATATGAATATCCTCCTTAG	*ihfA*
ihfA-H2P2		
ihfB-H1P1	TTGAAGGAAACCGGAGGAATAATGACCAAGTCAGAATTGATAGAATGTAGG CTGGAGCTGCTTCG GTTGTCGCCGCTCAGCGAAACTTACTCTTCGTAAATATTGGCGCGCATATG AATATCCTCCTTAG	*ihfB*
ihfB-H2P2		
*For mutant characterization*		
fis-MC-F	GCGCACATTCAACGCCATAG	*fis*
fis-MC-R	GCTTCCCCATGCCGAAGAG	
ihfA-MC-F	GAGGCATTGAAAGAGCGATTCC	*ihfA*
ihfA-MC-R	ACGGTATTCGGCGAAGAAAAG	
ihfB-MC-F	CTACGGCCGCCCTTGTTTAA	*ihfB*
ihfB-MC-R	TTAACGTTTAAGTATGTTGTCGCCG	
*For expression vector constructions*		
fis-NcoI-F	GAA***CCATGG***TCGAACAACGCGTAAATTCTGAC	*fis*
fis-HindIII-R	TGA***AAGCTT***TCAATGATGATGATGATGATGGTTCATGCCGTATTTTTTCAG	
ihfA-XhoI-F	GCA***CTCGAG***GAGCGATTCCAGGCATCATTG	*ihfA*
ihfA-EcoRI-R	GGG***GAATTC***ACGGTATTCGGCGAAGAAAAG	
ihfB-XhoI-F	GGG***CTCGAG***GCTAATTTTGCCTTGAAGGAAACC	*ihfB*
ihfB-EcoRI-R	GGG***GAATTC***TTACCACAACTTGCTGGCTG	
*For qPCR*		
aroX-F	TGTTGCCTGCAAGATTGACG	*aroX*
aroX-R	ATGTGTGAACGGCCAAAACG	
dhbX-F	ATGCGGCCAATCTGATGATG	*dhbX*
dhbX-R	AGCCCCAGAGCATAGGTAAATG	
icmX-F	TGATTGTCTGCGGCGTTTAC	*icmX*
icmX-R	GCTAGACGATGCTTTTCTTCGG	
adsX-F	TGCACATTGAACGGCAAGAC	*adsX*
adsX-R	ATCGAAGTGCAGGTTTCGTG	
hmoX-F	TCGCATGCCAAAGATTTCGC	*hmoX*
hmoX-R	ATGAGCTTGACGCGTTCAAC	
npsA-F	AAATACGTGGCTTCCGCATC	*npsA*
npsA-R	TCCTGCGTGACATAACAAGC	
thdA-F	TGGACAACGTTGAGCAACAG	*thdA*
thdA-R	TGCTTACCATTGACGCCAAC	
npsB-F	TGAGCATTTGCAGCTGGTTC	*npsB*
npsB-R	ATGCGTGGCAACTTTGTGTG	
rrsH-F	CAGCCACACTGGAACTGAGA	*rrsH*
rrsH-R	GTTAGCCGGTGCTTCTTCTG	
*For EMSA*		
aroX-npsA-F	TCTCTCACTCGAAATTTAACAGGT	*aroX-npsA*
aroX-npsA-R	TCTCTCCTGGAGAATTAGGAACG	
fbpA-F	TTCCTGACCAGCGAGCTGCCG	*fbpA*
fbpA-R	CCCCAGTACTCCCAGCTGTGC	

^a^The sequence corresponding to the template plasmid pKD4 is underlined. Italic/bold letters indicate the respective restriction enzyme site in the primer.

## Data Availability

The data presented in this study are available upon request from the corresponding authors.
